# Increased Activities against Biofilms of the Pathogenic Yeast *Candida albicans* of Optimized Pom-1 Derivatives

**DOI:** 10.3390/pharmaceutics14020318

**Published:** 2022-01-28

**Authors:** Valerie Amann, Ann-Kathrin Kissmann, Markus Krämer, Imke Krebs, Julio A. Perez-Erviti, Anselmo J. Otero-Gonzalez, Fidel Morales-Vicente, Armando Rodríguez, Ludger Ständker, Tanja Weil, Frank Rosenau

**Affiliations:** 1 Institute of Pharmaceutical Biotechnology, Ulm University, Albert-Einstein-Allee 11, 89081 Ulm, Germany; valerie.amann@uni-ulm.de (V.A.); ann-kathrin.kissmann@uni-ulm.de (A.-K.K.); markus-1.kraemer@uni-ulm.de (M.K.); imke.krebs@uni-ulm.de (I.K.); 2 Center for Protein Studies, Faculty of Biology, University of Havana, 25 Str. and I Str., La Habana 10400, Cuba; julio.perez@fbio.uh.cu (J.A.P.-E.); aotero@fbio.uh.cu (A.J.O.-G.); 3 General Chemistry Department, Faculty of Chemistry, University of Havana, Zapata y G, La Habana 10400, Cuba; femvicente@gmail.com; 4 Synthetic Peptides Group, Center for Genetic Engineering and Biotechnology, La Havana 10600, Cuba; 5 Core Facility for Functional Peptidomics, Ulm Peptide Pharmaceuticals (U-PEP), Faculty of Medicine, Ulm University, 89081 Ulm, Germany; armando.rodriguez-alfonso@uni-ulm.de (A.R.); ludger.staendker@uni-ulm.de (L.S.); 6 Core Unit of Mass Spectrometry and Proteomics, Faculty of Medicine, Ulm University, 89081 Ulm, Germany; 7 Max Planck Institute for Polymer Research Mainz, Ackermannweg 10, 55128 Mainz, Germany; weil@mpip-mainz.mpg.de

**Keywords:** antimicrobial peptides, *Candida albicans*, antibiofilm activity

## Abstract

Antimicrobial peptides (AMPs) are an alternative group for the therapy of infectious diseases, with activity against a wide range of diverse pathogens. However, classical AMPs have significant side effects in human cells due to their unspecific pore formation in biomembranes. Nevertheless, AMPs are promising therapeutics and can be isolated from natural sources, which include sea and freshwater molluscs. The AMPs identified in these organisms show promising antimicrobial activities, as pathogens are mainly fought by innate defence mechanisms. An auspicious candidate among molluscs is the Cuban freshwater snail *Pomacea poeyana*, from which the peptides Pom-1 and Pom-2 have been isolated and studied. These studies revealed significant antimicrobial activities for both AMPs. Based on the activities determined, Pom-1 was used for further optimization. In order to meet the emerging requirements of improved anti-biofilm activity against naturally occurring *Candida* species, the six derivatives Pom-1A to F were developed and investigated. Analysis of the derivatives acting on the most abundant naturally occurring *Candida* yeast *Candida albicans* (*C. albicans*) revealed a strong anti-biofilm activity, especially induced by Pom-1 B, C, and D. Furthermore, a moderate decrease in the metabolic activity of planktonic yeast cells was observed.

## 1. Introduction

**Figure 1:**
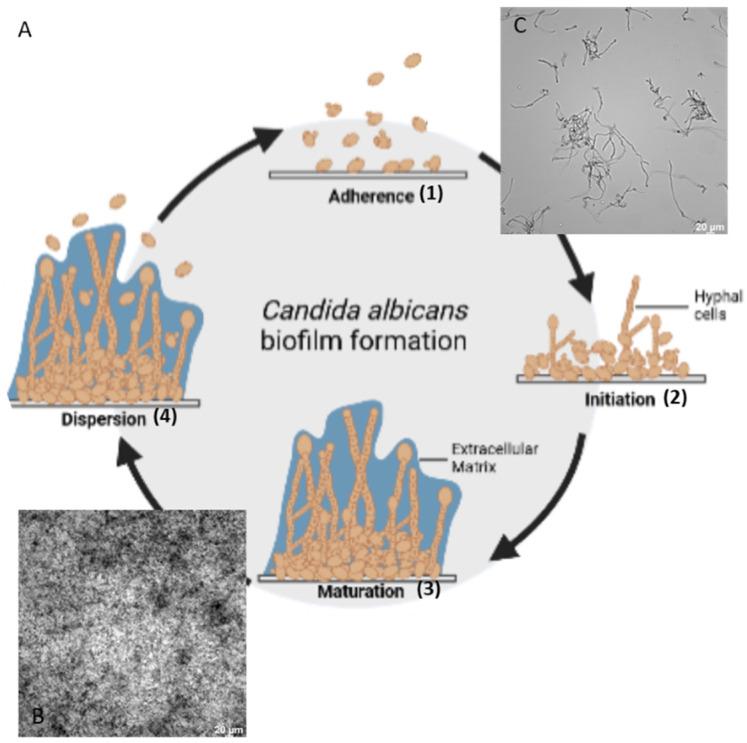
Overview of the biofilm formation of the yeast *C. albicans* structured in four steps (**A**). (1) Attachment of planktonic yeast cells to a surface, (2) aggregation and proliferation of cells, (3) formation of a mature biofilm with a species-specific ECM, (4) and detachment of yeast cells from the biofilm to form further biofilms. Micrographs photographed with transmission light in using a Leica DMi8 coded (Leica Microsystems CMS GmbH, Wetzlar, Germany) microscope after 24 h of growth at 37 °C. *C. albicans* biofilm (**B**) and planktonic cells (**C**).

A more than considerable amount of 75% of all women contract a fungal infection with the pathogenic genus *Candida*, like *C. parapsiolosis*, *C. glabrata,* or *C. tropicalis*, during their lifetime. In 85–95% of cases, these infections are caused by *C. albicans* [1]. In natural environments, many microorganisms, including yeast, form biofilms attached to abiotic and biotic surfaces. Yeasts adhere to surfaces, proliferate, and produce an extracellular matrix (ECM) forming microcolonies that mature (Figure 1). Cells disperse from the mature biofilms that adhere to surfaces and start a new cycle of biofilm formation [2]. This mechanism is a strategy of yeast cells to increase their physical stability to antifungal drugs, thus increasing the morbidity and mortality of infected patients [3,4,5,6,7].

Conventional treatment of *Candida* infections is based on (targeted) pharmaceutical therapy using compounds like the azole antimycotic fluconazole, which inhibits the enzyme 14-α-demethylase and thus prevents the formation of ergosterol, which is responsible for membrane stability [8].

However, since 2016, the Clinical Practice Guideline from Management of Candidiasis from the Infectious Disease Society of America no longer recommends the use of fluconazole as first-line treatment for less sensitive strains of the genus *Candida*, as these yeasts can often quickly develop high tolerances [9,10]. Several mechanisms, such as binding of the azole to β-1,3-glucan at the ECM and its exclusion by azole efflux pumps or mutations in the structure or expression of the target protein, have been identified to be decisive factors triggering resistance [11,12,13,14,15]. Due to these characteristics, resistant *Candida* species are classified as a “serious threat” by the CDC of the U.S. Department of Health and Human Services (Centers for Disease Control and Prevention (CDC), 2019) [16].

**Figure 2:**
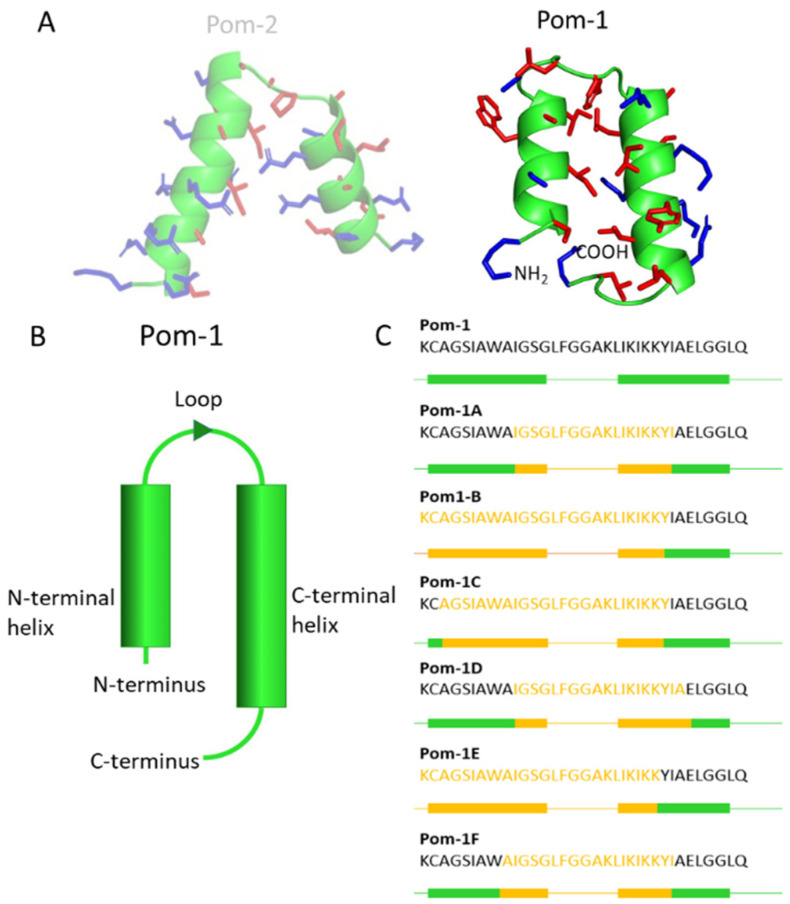
Representation of the Pom-1 and Pom-2 (in transparent, as not used further in this study) structure by a ribbon model with corresponding side chains (**A**) and schematic ribbon model modeled with QUARK and SwissModel (**B**), as well as the amino acid sequence with schematic representation of the α-helical structure of Pom-1 (green) in comparison to the optimized (i.e., truncated) derivatives Pom-1A to F (yellow) (**C**). In this illustration, the boxes indicate the α-helices, while the lines represent the loops.

Antimicrobial peptides (AMPs) may represent an alternative to these conventional drugs sd they do not bind specifically to a protein of the pathogen to inhibit its enzymatic activity, but can modify their physical or chemical properties and alter the membrane structure and the physiological integrity of the pathogen. The key factor for this efficacy lies in their intrinsic amphiphilic character, normally with both cationic and hydrophobic residues along the peptide sequence [17,18]. As these peptides are found in virtually all life forms, nature appears to offer an almost unlimited diversity that can provide medicine with promising new molecules for the upcoming area of short running potent classical antibiotics and antimycotics. Sea- and fresh-water molluscs, which have been identified as one of the most diverse phyla on Earth, carry these AMPs in their innate immune system to fight off viruses, bacteria, yeasts, and parasites [19]. The AMPs Pom-1 and Pom-2 were isolated from the freshwater snail *Pomacea poeyana* (Pilsbry, 1927), and show an α-helical structure in a membrane-like environment [1] (Figure 2). These peptides have been shown to be effective not only against the pathogenic bacteria *Pseudomonas aeruginosa*, *Klipsella pneumonia*, and *Listeria monocytogenes* [20], but also with low activity against planktonic and high activity cells of yeasts of the genus *Candida*. Pom-1 was used for optimization as it exhibited a higher antimicrobial activity than Pom-2 [21].

**Figure 3:**
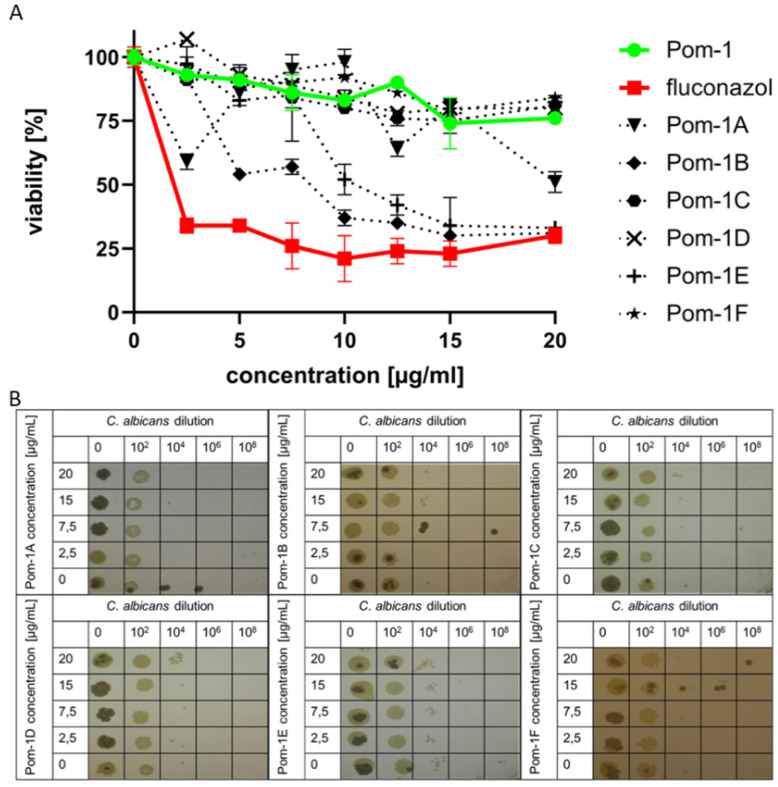
Antifungal activity of Pom-1A to F towards planktonic cells of *C. albicans*. (**A**) Determined dose-dependent effect of Pom-1 derivatives on cell viability by resazurin-reduction assay with fluconazole and Pom-1 as the control agents. All experiments were performed in triplicate. (**B**) Plate spot assay after 24 h incubation of *C. albicans* cultures with defined cell number at various concentrations of Pom-1A to F. 10^2^, 10^4^, 10^6^, and 10^8^-fold dilutions of the original culture were added to agar and incubated for an additional 24 h at 37 °C.

**Figure 4:**
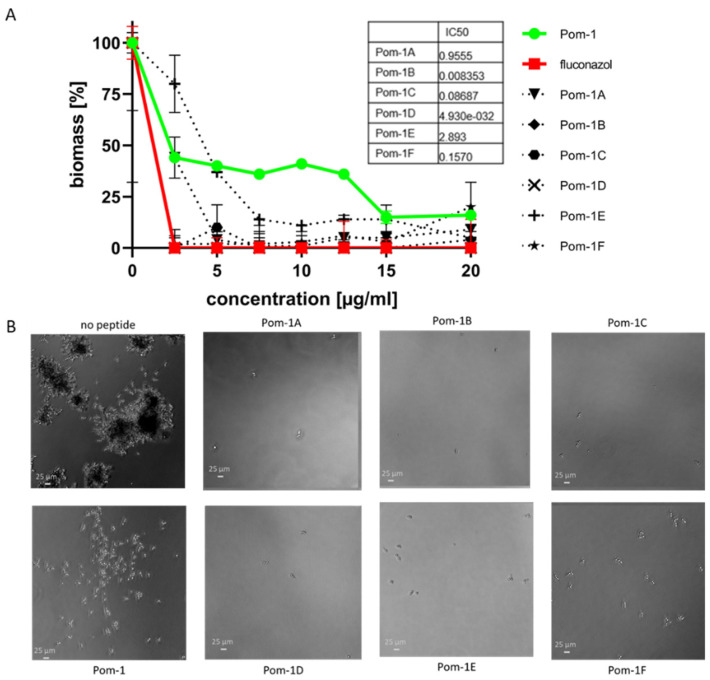
Antifungal activity of Pom-1A to F towards *C. albicans* biofilms. (**A**) Determined dose-dependent effect of Pom-1 derivatives on biofilm mass by crystal violet assay with fluconazole and Pom-1 as the control agent. All experiments were performed in triplicate and error bars indicate standard deviations. (**B**) Microscopic analysis using a Leica DMi8 coded (Leica Microsystems CMS GmbH, Wetzlar, Germany) microscope) of untreated *C. albicans* biofilm and biofilm treated with Pom-1 and its derivatives (20 µg/mL) after 24 h of incubation at 37 °C under transmitted light.

This peptide is a 34-amino acid-long fragment of Closticin 574, a bacteriocin with a known antibacterial activity against several *Clostridium* spp. [22]. According to structure prediction (QUARK and SwissModel), Pom-1 consists of two α-helices (shorter N-terminal α-helix with predominantly hydrophobic properties and longer C-terminal α-helix with amphiphilic properties), which are connected by a short loop. Based on this peptide, six derivatives, Pom-1A to F (Figure 2), were selected after in silico generation from Pom-1 and further ranking according to the highest AMP prediction probability (no specific AMP activity/effect was further predicted). In Figures 3 and 4, it is shown that while Pom-1B and Pom-1E have a higher inhibitory effect on planktonic cells of *C. albicans* than Pom-1, all peptides have a better antifungal effect on biofilm formation, while Pom-1B, C, and D have an extraordinary reduction in the biofilm formation of *C. albicans.* In addition, only marginal cytotoxicity against human dermal fibroblasts (HDF) [23] and adenocarcinomic human alveolar basal epithelial cells (A549) [24] was detected with these three peptides.

## 2. Materials and Methods

### 2.1. Materials

Acetic acid, agar−agar, crystal violet, glucose, 3-(N-morpholino) propanesulfonic acid (MOPS), peptone, and yeast extract were ordered from Carl Roth GmbH (Karlsruhe, Germany), and RPMI-1640 medium supplemented with L-glutamine was purchased from Thermo Fisher Scientific (Waltham, MA, USA). Fluconazole was obtained from Merck KGaA (Darmstadt, Germany) and resazurin sodium salt was sourced from Sigma-Aldrich Chemie GmbH (Steinheim, Germany). Dulbecco’s modified eagle medium (DMEM) supplemented with fetal bovine serum (FBS; 10% (*w*/*v*)), penicillin-streptomycin (100 U mL^−1^, 1% (*w*/*v*)), eagle’s minimum essential medium non-essential amino acids (MEM NEAA) (1% (*w*/*v*)), and accutase^®^ solution were ordered from Life Technologies (Carlsbad, CA, USA). Phosphate-buffered saline (PBS) was sourced from Life Technologies (Carlsbad, CA, USA).

### 2.2. Methods

#### 2.2.1. Cultivation of C. albicans

*Candida albicans* (ATCC 90028) was sourced from the IPK Medical Mycology Laboratory and cultured on Sabouraud dextrose agar (40 g/L glucose, 10 g/L peptone, 20 g/L agar, pH 5.6). For the suspension cultures, 10 mL of RPMI-1640 medium supplemented with L-glutamine (Thermo Fisher Scientific (Waltham, MA, USA)) was inoculated with a single colony in a 100-mL Erlenmayer flask and was grown at 37 °C with orbital shaking at 150 rpm for 16 h [21].

#### 2.2.2. Peptide Optimization

Pom-1 fragments (10 or more aa residues) were generated in silico by a Protein Digestion Simulator, https://pnnl-comp-mass-spec.github.io/Protein-Digestion-Simulator/ from PNNL and the Protein-Digestion-Simulator GitHub repository (accessed on 10 February 2021) [25], and were then evaluated by the CAMPR3-Predict Antimicrobial Peptide tool http://www.camp.bicnirrh.res.in/predict/ (accessed on 10 February 2021) [26], Peptide AMP Scanner, https://www.dveltri.com/ascan/v2/ascan.html (accessed on 10 February 2021) [27] and iAMPpred, http://cabgrid.res.in:8080/amppred/server.php (accessed on 10 February 2021) [28]. The results were merged and the AMP prediction probability (no specific AMP activity/effect was further predicted) values from all servers were averaged for every sequence. A rank list was organized by decreasing order of AMP prediction probability (Table S1). Six peptides (Pom-1A to F) with the highest values were synthesized automatically in a 0.10 mmol scale using standard Fmoc solid phase peptide synthesis techniques with the microwave synthesizer Liberty blue (CEM GmbH, Kamp-Lintfort, Germany), as described previously [20].

#### 2.2.3. Resazurin-Reduction-Assay/Viability-Assay

The viability of treated *C. albicans* cells with Pom-1A to F was determined according to the Clinical and Laboratory Standards Institute guidelines (M27-A3) for the broth microdilution assay [29]. For this, 2.5 × 10^3^ yeast cells were incubated in 200 µL of RPMI-1640 medium (supplemented with L-glutamine) in the presence of various concentrations of Pom-1A to F (2.5–20 µg/mL) in flat-bottomed polystyrene microtiter plates with 96 wells (Sarstedt AG and Co. KG, Nümbrecht, Germany) at 37 °C with shaking at 900 rpm on an Eppendorf shaker. Quantitation of viable yeast cells was performed using a Resazurin-reduction-Assay [30]. For this, yeast cells were incubated with 20 µL of 0.15 mg/mL resazurin solution for 2 h. During this time, the viable cells were able to reduce the resazurin to resorufin. With fluorescence measurements at an excitation wavelength of 535 and an emission of 595 nm with a Tecan infinite F200 microplate reader (Tecan Group Ltd., Männedorf, Switzerland) the amount of converted resorufin could be determined, and thus the viability of the cells could be quantified. The data obtained were subsequently identified in a Hill nonlinear dose-response equation and the semi-inhibitory concentration (IC_50_) could be determined, at which the cell viability was reduced by 50% compared to the untreated control. In addition, after incubation, 1 µL cell suspension was taken from each well of the microtiter plate to visually confirm the graphical results in a plate spot assay [31]. For this purpose, a dilution series was prepared for each sample in RPMI 1640 medium and 1 µL was added to Sabouraud agar plates, which were then incubated for 24 h at 37 °C, respectively.

#### 2.2.4. Biofilm Formation and Crystal-Violet-Assay/Biomass-Quantification

The analysis of the antifungal effect of the peptides on biofilm followed the same conditions as described previously [32,33]. In brief, 2.5 × 10^3^ yeast cells were incubated in 200 µL RPMI-1640 medium with L-glutamine in flat-bottomed polystyrene microtiter plates with 96 wells (Sarstedt AG and Co. KG, Nümbrecht, Germany) with the respective peptides at different concentrations (2.5–20 µg/mL) for 24 h at 37 °C, but without agitation. The quantification of the biofilm took place due to a crystal violet assay, which was originally developed by George O’ Toole for bacteria [32,34] and was adapted for Candida biofilms [5,32,34,35]. For this, the planktonic phase in the wells was removed and the biofilms were washed twice with 200 µL of demineralized water. Subsequently, the cells in the biofilm were stained for 15 min with 200 µL of a 0.1% (*w*/*v*) crystal violet solution. The solution was then removed again and the stained cells were washed twice with 200 µL of demineralized water. Then, the biofilms in the microtiter plate were dried for 24 h at 25 °C. These were then mixed with 200 µL of 30% acetic acid. After 15 min, the stained solution was transferred to a new microtiter plate and the absorbance at 560 nm was measured using a Tecan infinite F200 microplate reader. The data obtained were then graphically identified using a Hill non-linear dose-response equation and the semi-inhibitory concentration (IC_50_) was determined, at which 50% of the biofilm was not formed compared to the untreated control.

#### 2.2.5. Cell Culture

Adenocarcinomic human alveolar basal epithelial cells A549 [24] and human dermal fibroblasts HDF [23] were used for the experiments. The cultivation of these cells was performed in DMEM supplemented with FBS (10% (*w*/*v*)), MEM NEAA (1% (*w*/*v*)), and penicillin-streptomycin (100 U mL-1, 1% (*w*/*v*)) in a 37 °C incubator containing 5% CO_2_.

#### 2.2.6. Passaging Adherend Cell Cultures

Accutase was used to seed the adherent cell cultures. First, the medium was preheated with additives at 37 °C. Then, the medium in the culture flask was removed and 2.5 to 5 mL accutase was immediately added. The flask with accutase was incubated for 5 to 10 min at room temperature until the cells had formed a rounded shape. For detaching the cells from the ground, the culture flask was knocked against the back of the hand. A desired cell number was aliquoted into a new culture flask containing 5 mL DMEM medium with supplements and incubation at 37 °C containing 5% CO_2_.

#### 2.2.7. Viability-Assays for Cell Cultures

A resazurin assay was performed to detect cell viability. For this, 2 × 10^4^ cells per well were incubated for 48 h in a 96-well-plate in DMEM with supplements (100 µL) and different peptide concentrations (2.5 µg/mL, 20 µg/mL) at 37 °C containing 5% CO_2_. Afterwards, 20 µL resazurin (0.15 mg/mL) was added to each well and was incubated for 24 h at 37 °C containing 5% CO_2_. Thereafter, the fluorescence of resorufin was measured with a Tecan infinite F200 microplate reader (Tecan Group Ltd., Männedorf, Switzerland) at an excitation wavelength of 535 nm and an emission wavelength of 595 nm.

#### 2.2.8. ExPASy ProtParam

To determine the peptide properties of the Pom-1 derivatives, ProtParam analysis tool (Expasy) was used [36]. This tool calculates the GRAVY (Grand average of hydropathicity) (Formula 1) and the Amphihilic Index (Formula 2) of the desired peptide according to the following formulas.

(1) $$\mathrm{GRAVY}=\frac{\mathrm{sum}\;\mathrm{of}\;\mathrm{hydropathy}\;\mathrm{values}\;\mathrm{of}\;\mathrm{all}\;\mathrm{the}\;\mathrm{amino}\;\mathrm{acid}}{\mathrm{number}\;\mathrm{of}\;\mathrm{resedues}\;\mathrm{in}\;\mathrm{the}\;\mathrm{sequence}\;}$$

(2) Amphipilic Index=X(Ala)+a×X(Val)+b×[X(Ile)+X(Leu)]

To determine the amphiphilic index, the mole percentages (*X*) of the amino acids alanine (Ala), valine (Val), isoleucine (Ile), and leucine (Leu) were added together, taking into account the relative volume of valine side chains (a = 2.9) and Leu/Ile side chains (b = 3.9) of Ala.

#### 2.2.9. Statistical Analysis

With Student’s *t*-test, the statistical significance was tested. *p* values < 0.05 were considered significant, * denotes *p* < 0.05, ** *p* < 0.01 and *** *p* < 0.001. Standard deviations were represented as error bars.

## 3. Results

### 3.1. Pom-1 A-F Are Active against Planctonic Cells

To determine the antifungal effect of the peptide derivatives on planktonic *C. albicans* cells, different concentrations (2.5–20 µg/mL) of the peptides were applied to fixed cell numbers (2.5 × 10^3^ cells per well) and were incubated for 24 h in a suspension microtiter plate. A resazurin reduction assay was then performed to determine the cell viability, where the growth of the untreated cell was used as the reference value.

The results of the resazurin reduction assay for the treated planktonic *C. albicans* cells with Pom-1, Pom-1A to F, and fluconazole did not show 100% inhibition of cell viability for any agent in the concentration range of 0–20 µg/mL (Figure 3A). The peptide Pom-1B and Pom-1E, similar to the commercially used fungicide fluconazole, showed up to 70% inhibition of cell viability at a concentration of 20 µg/mL. Furthermore, Pom-1A showed approximately 50% inhibition of cell viability at the same concentration, while all other derivatives (Pom-1C, D, and E), as well as Pom-1, did not even reach this 50% inhibition of *C. albicans* cell viability.

To confirm these results, a plate spot assay was performed on *C. albicans* planktonic cells treated with the different peptides (Figure 3B). For this purpose, 1 µL cell suspension was taken from each well before adding the resazurin solution from the suspension microtiter plate. This sample was then diluted and was added to a Sabourand agar plate, which was then incubated for an additional 24 h for colony formation.

Here, we observed that colony formation decreased with the increase of peptide concentrations for Pom-1A, Pom-1B, and Pom-1D, and the use of higher dilutions of *C. albicans* samples, whereas Pom-1C, Pom-1D, and Pom-1F again showed only marginal colony formation.

### 3.2. The Novel Peptides Inhibit C. albicans Biofilm-Formation

The Pom-1 wildtype peptide showed a significant activity against *C. albicans* biofilms [21]. In order to evaluate the potential of the novel Pom-1A-F peptides, the well established crystal violet assay for microbial cultures in microtiter plates was performed to quantify the biomass formed.

In contrast to their moderate activity against planktonic *C. albicans* cells in the tested concentration range, Pom-1A to F application resulted in up to 100% inhibition of biofilm formation already at low concentrations of the peptides. While the original Pom-1 peptide has a semi inhibitory concentration (IC_50_) of 3.8, the derivatives had significantly lower values, which were very similar to the antifungal effect of fluconazole (IC_50_ 4.9e-032) (Figure 4). Pom-1B (IC_50_ 0.008353), Pom-1C (IC_50_ 0.08687), and Pom-1D (IC_50_ 4.9e-032) showed 100% inhibition of biofilm formation starting at a concentration of 2.5 µg/mL, whereas Pom-1 did not even show 100% inhibition in the concentration range assayed. All other peptide derivatives (Pom-1A, E, and F) also did not show 100% inhibition in the concentration range tested, but they still exhibited a higher antifungal activity against *C. albicans* biofilm than the original Pom-1 peptide, calculated as IC_50_ of 0.9555 for Pom-1A, 2.893 for Pom-1E, and 0.1570 for Pom-1F.

Microscopic analysis confirmed these results. *C. albicans* biofilms were treated with Pom-1, and its derivatives in comparison to an untreated bifilm control (Figure 4B). The latter control biofilm showed the highest density on the surface substratum, whereas a significant reduction was observed when the biofilms were formed in the presence of the Pom-1 wild type peptide. Compared to these control biofilms, Pom-1A to D showed a very similar cell density, whereas a slightly higher surface coverage was present for cultures incubated with Pom-1E and Pom-1F.

In summary, the results of the antifungal activity of the Pom-1 derivatives on planktonic and biofilm cells of *C. albicans* together with their physicochemical properties are shown in Table 1.

If results of the experiments (Figure 3 and Figure 4) are compared to the peptide properties of the Pom-1 derivatives determined via ExPASy ProtParam (Table 1), it can be observed that there is no association between the amphiphilic character of the peptides and their antifungal activity. Compared to Pom-1 (assumed as 100%), Pom-1B showed the lowest (89%) and Pom-1D (113%) the highest amphiphilic index compared to the six derivatives, and both of them are the most effective derivatives against biofilm formation.

### 3.3. Viability of Cell Culture

**Figure 5:**
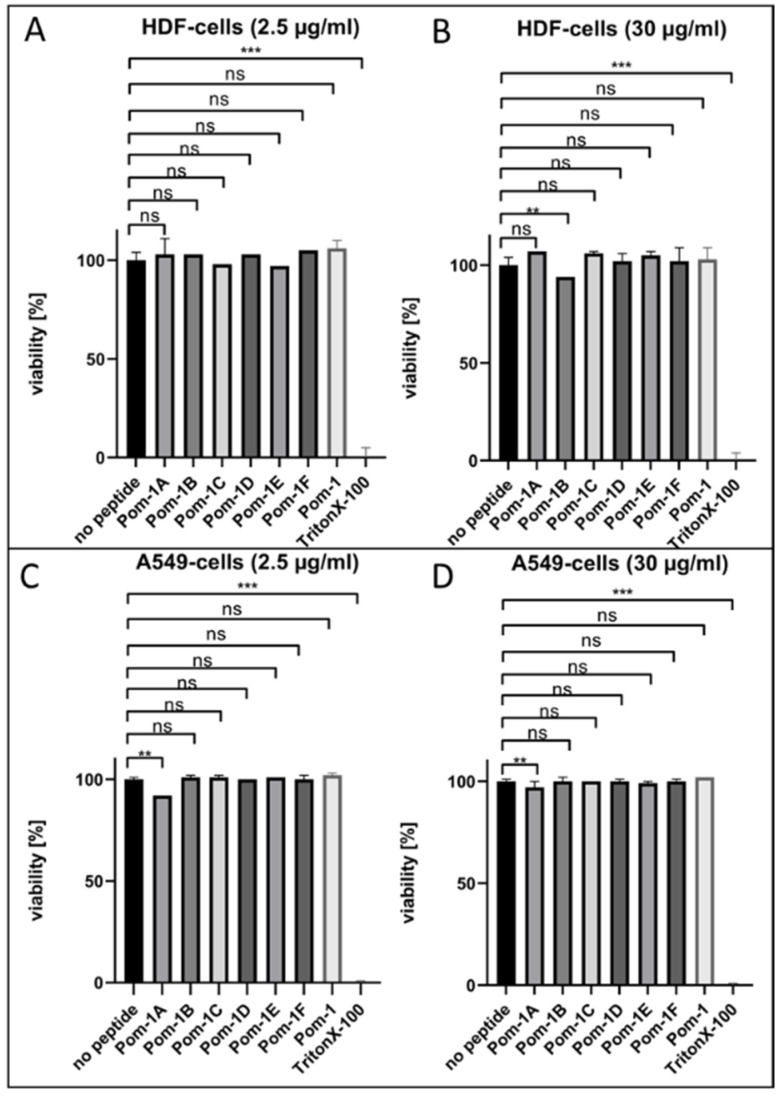
Analysis of cell viability for HDF and A549 cell lines without reagents and after the addition of Pom-1 derivatives, Pom-1 and Triton X-100. (**A**) Cell viability after the addition of 2.5 µg/mL of the respective reagent to HDF cells. (**B**) Cell viability in the presence of 30 µg/mL of the respective reagent to HDF. (**C**) Cell viability after application of 2.5 µg/mL of the respective reagent to A549 cells. (**D**) Cell viability after treatment with 30 µg/mL of the respective reagent to A549 cells. *p* values < 0.05 were considered as significant. ns denotes not siginificant; ** denotes *p* < 0.01; *** denotes *p* < 0.001.

As an important property determining the application potential of AMP, the cytotoxicity to human cells was initially tested by incubating Adeno carcinomic human alveolar basal epithelial cells A549 [24] and human dermal fibroblasts HDF [23] cell lines with all Pom-1 derivatives for 24 h. These cells were used based on their easy handling (A549) and to test whether it would be possible to use these peptides in combination with wound dressings for further experiments in a basic skin model (HDF). Three controls served as the reference in these experiments. The viability of untreated cells was measured, as well as cell viability after treatment with the original Pom-1 and surfactant Triton X-100 with known cytotoxicity added at concentrations of 2.5 µg/mL and 30 µg/mL. A resazurin reduction assay was then performed to determine the cell viability, and the fluorescence of the reduction product resorufin was measured (Figure 5). The evaluation of the cytotoxicity was expressed as percent of viability. For this purpose, the measured values of the controls without reagents were taken as 100%.

These experiments showed only marginal reductions of cell viabilities compared to the untreated samples for the peptides Pom-1A starting at a concentration of 2.5 µg/mL on A549 cells (Figure 5C,D), and Pom-1B starting at a concentration of 30 µg/mL on HDF cells (Figure 5B). All other derivatives including the original peptide Pom-1 did not show any reduction in cell viability at the concentrations tested.

## 4. Discussion

As clinical *C. albicans* isolates continuously increase their tolerance against various commercially used antimycotics, alternatives must be sought that support or even replace the commercially used therapeutic pathways. Meanwhile, clinical strains of *C. albicans* reach a minimum inhibitory concentration (MIC) of 0.5 to 64 µg/mL to fluconazole, 0.03 to 16 µg/mL to intraconazole (with these strains being considered resistant from 1 µg/mL), and 0.125 to 4 µg/mL to amphotericin-B, which is considered as the golden standard for candidiasis [9,37,38,39]. Furthermore, treatment with these antifungals is associated with many side effects, according to the National Health Service of the United Kingdom. Thus, treatment with fluconazole often leads to headache, stomach pain, diarrhea, nausea, and vomiting, as well as skin rash [40]. When azoles are used for weeks to months after stem cell transplantation, for example, they can even lead to hepatotoxicity and hormonal side effects such as impotence, as well as muscle and nerve disfunction. Other known side effects of the azole drug group include pancreatitis, phototoxic reactions, and peripheral neuropathy [41]. In addition, amphotericin-B is nephrotoxic at high doses and can cause nausea, vomiting, fever, hypoxia, and other side effects [42]. Thus, every molecule added to the portfolio of promising antifungal drugs in the fight against strains with pronounced resistance against the traditional compounds can be regarded as an important step towards possible treatment strategies involving the novel drugs in combination with the standard molecules to reduce the required high concentrations and thus their severe side effects.

The antifungal effect of the wildtype Pom-1 peptide against planktonic *C. albicans* cells could be slightly improved by the alterations introduced with the derivatives Pom-1A, Pom-1B, and Pom-1E, which have similar activities like the reference fungicide fluconazole in the tested concentration range (0–20 µg/mL). All derivatives, more importantly, represented considerable or impressive improvements of the inhibition of *C. albicans* biofilm formation (Table 1).

Although the exact molecular mechanisms behind AMP activities is accepted to be currently still not comprehensively understood, two main ways of action are in discussion [43,44,45]. In recent years AMPs appeared to be active intracellularly, which were considered to exert their antimicrobial activity by binding to specific targets within the cell [46]. On the other hand, there are the more classical membrane-active AMPs. This group is thought to have only activities based exclusively on their physicochemical properties, which are suited to assemble into macromolecular structures that interact with biomembranes to destabilize them and to compromise their full structural and functional integrity forming nano-scale pores [47]. The key factors in this mechanism are thought to have been identified as the cationic, amphiphilic, and hydrophobic character of the respective AMPs [17,18]. Due to their high hydrophobicity and amphiphilicity, as well as the low cationic character of the peptides, they can interact well with the negatively charged but overall hydrophobic pathogen membrane [46].

Bioinformatic analysis showed an improvement in the amphiphilic index for derivatives A, D, and F, and an improvement in the GRAVY value for all derivatives except Pom-1B (Table 1).

Based on our results, it is reasonable to suspect that the Pom-1 derivatives do not act like conventional membrane-active AMPs, but more like Cm-p5 derivatives, which we have recently described to probably act via a novel mechanism at the membrane [48]. As the peptides are only moderately effective against planktonic cells, but are very effective at preventing biofilm formation, we assume that the peptides likely do not form pores, but rather aggregate on the pathogenic membrane (“carpet-model” [46]), thereby preventing biofilm formation. To suspect a different mode of action is also supported by the fact that Pom-1 and also its derivatives show only marginal cytotoxic effects on human cells, suggesting that the simple physical formation of harmful pores in the biomembranes is not the key effect. To elucidate the molecular mechanism behind this potential cell−cell and/or cell−substrate interaction disturbed by the (peptide) complexes will be part of in-depth follow up studies. Nevertheless, with an improvement of anti-biofilm activity against *C. albicans* of several orders of magnitude for Pom-1B, C and D, we developed peptides that are similarly effective to fluconazole as one of the traditional standard therapeutics. Although the extent of the development of potential resistance against these peptides needs to be carefully determined, we believe that their tendency to be selective towards resistance can be expected to be moderate. Thus, these novel peptides maybe a promising starting point for further optimization, and thus may represent attractive lead structures on the way towards potent future peptide-based anti-*C. albicans* therapeutics.

## 5. Conclusions

Classic antifungal drugs have many negative side effects and long-term therapy is often required [40,47]. As antifungal resistance is increasing, higher concentrations are applied and aggravate this problem. Small AMPs with a high antifungal activity and low cell toxicity could therefore be a strong resource in the fight against candidiasis. In this study, we found out that optimization of the naturally occurring AMP Pom-1 by shortening of the original sequence improved antifungal activity against planktonic cells, as well as against biofilm formation of the yeast *C. albicans*, which causes up to 95% of all candidiasis [1]. While derivatives Pom-1B to D show an excellent antifungal activity against the biofilm formation of *C. albicans*, only Pom-1B and Pom-1E show a slightly improved antifungal activity against planktonic cells on *C. albicans* yeast cells in comparison to Pom-1. Based on the low cytotoxicity, it is assumed that these peptides do not develop their antimicrobial activity by pore formation like conventional AMPs, but according to the “carpet model” [46], and bind to specific targets on the pathogenic membrane and thus prevent cell−cell interaction and biofilm formation. This assumption creates the incentive to conduct further studies to provide the exact mechanism of action of these novel AMPs.

For further optimization, such as the generation of a cyclic version or a multivalent peptide, Pom-1B could be used considering its structural features. Moreover, it is not yet known how these peptide derivatives act in combination with each other or with conventional drugs.

## Figures and Tables

**Table 1:** Overview of the peptide properties calculated with ExPASy ProbParam [36] (peptide length, truncation of derivatives sequence compared to Pom-1 starting from C- and N-terimus, amphiphilic index, GRAVY (grand average of hydropathicity)), as well as the antimicrobial activities (against the planktonic cells at 20 µg/mL peptide concentration and biofilm based on IC_50_ values of the pathogenic yeast *C. albicans*) of the derivatives in comparison to Pom-1 expressed in percent and the IC_50_ values of these peptides against biofilm formation of *C. albicans*.

Peptide	Length [AS]	Truncation	Amphipilic Index [%]	GRAVY [%]	IC_50_ Values against Biofilm Formation	Antimycrobial Activity against Planktonic *C. albicans* Cells [%] at 20 µg/mL	Antimicrobial Activity against *C. albicans* Biofilm [%] Based on IC_50_ Values
Pom-1	34		100	100	3.8	100	100
Pom-1A	18	N–9; C–7	115	109	0.95	133	400
Pom-1B	26	C–8	89	89	0.008	160	47500
Pom-1C	24	N–2; C–8	97	107	0.08	100	4750
Pom-1D	19	N–9; C–6	113	120	4.93 × 10^−32^	97	8 × 10^33^
Pom-1E	25	C–9	93	101	2.89	153	131
Pom-1F	19	N–8; C–7	113	120	0.16	82	2375

## Supplementary Materials

The following supporting information can be downloaded at: www.mdpi.com/article/10.3390/pharmaceutics14020318/s1, Table S1: Six peptides (Pom-1A to F).
